# Development of a luminescent G-quadruplex-selective iridium(III) complex for the label-free detection of adenosine

**DOI:** 10.1038/srep19368

**Published:** 2016-01-18

**Authors:** Lihua Lu, Hai-Jing Zhong, Bingyong He, Chung-Hang Leung, Dik-Lung Ma

**Affiliations:** 1 Department of Chemistry, Hong Kong Baptist University, Kowloon Tong, Hong Kong, China; 2 State Key Laboratory of Quality Research in Chinese Medicine, Institute of Chinese Medical Sciences, University of Macau, Macao, China; 3 Partner State Key Laboratory of Environmental and Biological Analysis, Hong Kong Baptist University, Hong Kong, China

## Abstract

A panel of six luminescent iridium(III) complexes were synthesized and evaluated for their ability to act as G-quadruplex-selective probes. The novel iridium(III) complex **1** was found to be highly selective for G-quadruplex DNA, and was employed for the construction of a label-free G-quadruplex-based adenosine detection assay in aqueous solution. Two different detection strategies were investigated for adenosine detection, and the results showed that initial addition of adenosine to the adenosine aptamer gave superior results. The assay exhibited a linear response for adenosine in the concentration range of 5 to 120 μM (R^2^ = 0.992), and the limit of detection for adenosine was 5 μM. Moreover, this assay was highly selective for adenosine over other nucleosides, and exhibited potential use for biological sample analysis.

Adenosine, an endogenous purine nucleoside, is involved in many physiological processes in various tissues and organs^1^. In the peripheral nervous system, adenosine plays an important role in the regulation of muscle contraction, cerebral and blood flow, and is an effective vasodilator. In the central nervous system, it is involved in the modulation of neurotransmission and acts as a neuroprotective agent against hypoglycemia, hypoxia, and ischemia^2^,^3^. Moreover, adenosine has an inhibitory effect on synaptic activity and slows down the metabolic activity of the brain, and may also play an important role in the immune system^4^.

Therefore, it is of great importance to develop simple and rapid detection methods for adenosine. Chromatographic techniques including high-performance liquid chromatography (HPLC)^5^ and gas/liquid chromatography-mass spectrometry (GC/LC-MS)^6^ have been widely employed to detect adenosine. However, these techniques tend to require expensive instrumentation, tedious preparation procedures, and trained operators. On the other hand, aptamers are nucleic acid sequences that can selectively bind to specific molecules, and which have been widely employed for bioanalytical applications^7^. Szostak and co-workers reported that the 27-mer adenosine aptamer binds to adenosine with a dissociation constant of 6 μM^8^, and this binding event is accompanied by a transition of the aptamer from a random single-stranded DNA (ssDNA) conformation into a hairpin-like conformation. The “structure-switching” response of the adenosine aptamer has been transduced into colorimetric^9^,^10^,^11^,^12^,^13^, luminescent^3^,^7^,^14^,^15^,^16^,^17^,^18^, electrochemical^4^,^19^,^20^,^21^,^22^,^23^,^24^,^25^,^26^, electrochemiluminescent (ECL)^27^,^28^,^29^,^30^ or surface-enhanced Raman scattering (SERS)^31^,^32^,^33^,^34^ signals in various adenosine detection assays. However, the majority of these aptamer-based assays for adenosine detection required labeled oligonucleotides and/or multiple DNA probes^35^,^36^,^37^,^38^.

The G-quadruplex structure, a non-canonical DNA secondary structure consisting of planar stacks of four guanines stabilized by Hoogsteen hydrogen bonding, has drawn increasing interest in various analytical applications^14^. To construct label-free G-quadruplex-based detection platforms, it is important to develop probes that possess high affinity and selectivity for G-quadruplex DNA. However, some commonly-used organic dyes, such as crystal violet, thiazole orange and thioflavin T, show promiscuous binding to multiple DNA conformations^39^. Meanwhile, transition metal complexes have attracted great interest as probes for biomolecules due to their long lifetimes, large Stokes shifts, simple synthetic protocols and tunable excitation and emission maxima over the visible region^40^,^41^,^42^,^43^,^44^,^45^. In particular, iridium(III) octahedral complexes have been recently studied as G-quadruplex-selective probes for the development of luminescent assays^46^,^47^. Recently, our group has developed a variety of iridium(III) complexes as G-quadruplex-selective probes for the construction of a range of label-free luminescent detection platforms^48^,^49^,^50^,^51^. In this work, we envisaged that we could develop a G-quadruplex-selective iridium(III) complex for use in the construction of a label-free, aptamer-based luminescent switch-on detection platform for adenosine.

## Results

**Figure 1:**
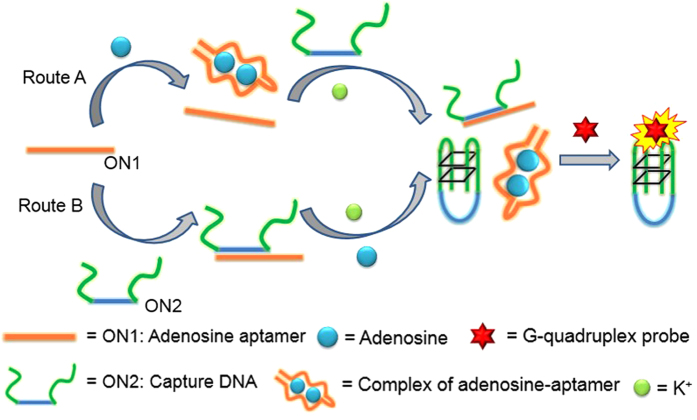
Schematic presentation of the aptamer-based adenosine detection by using a G-quadruplex selective luminescent probe showing experimental detection strategies Route A and Route B.

The proposed mechanism of the aptamer-based detection platform for adenosine using the luminescent G-quadruplex-selective Ir(III) probe is depicted in Fig. 1. In this work, two different detection strategies, Route A and Route B, were investigated. In Route A, the adenosine aptamer ON1 (5′-AC_2_TG_5_AGTAT_2_GCG_2_AG_2_A_2_G_2_T-3′) is first reacted with adenosine to generate an adenosine-aptamer complex. Then an equimolar concentration of the capture DNA ON2 (5′-G_3_T_3_G_3_ACTC_5_AG_2_TG_3_T_3_G_3_-3′), which is partially complementary to ON1 and which also contains two halves of a split G-quadruplex at its two termini, is added to the reaction mixture. Some of the ON2 hybridizes with the free ON1 in solution, and this prevents the split G-quadruplex-forming sequences of ON2 from coming into proximity and forming a G-quadruplex motif. Meanwhile, the remaining, unhybridized ON2 can be induced into a G-quadruplex structure in the presence of K^+^ ions, and which can be bound by the iridium(III) complex with an enhanced luminescent response. The greater the concentration of adenosine initially present in the solution, the more adenosine-ON1 complexes are formed, and hence the more unhybridized ON2 are available for G-quadruplex formation. Therefore, this system functions as a label-free switch-on luminescent detection platform for adenosine.

In Route B, the adenosine aptamer ON1 and the capture DNA ON2 are initially hybridized to form a duplex substrate ON1-ON2. Then the addition of adenosine causes ON1 to dissociate from ON2, allowing the released ON2 to fold into a G-quadruplex motif in the presence of K^+^ ions. This G-quadruplex is similarly recognized by the G-quadruplex-selective probe with an increased luminescent response.

Both routes rely on the fact that the binding affinity of aptamer ON1 toward adenosine is stronger than that for the partially complementary capture DNA ON2. Consequently, in Route A, ON2 is unable to dissociate already-formed adenosine-ON1 complexes, whereas in Route B, adenosine can dissociate pre-formed ON1-ON2 duplexes.

**Figure 2:**
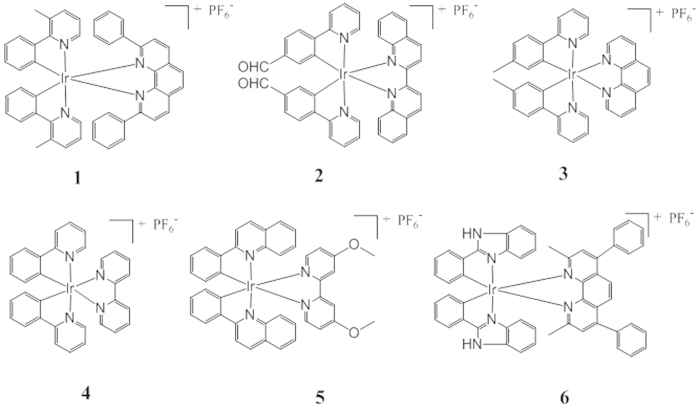
Chemical structures of Ir(III) complexes (**1–6**) used in this study. Complex (**1**), [Ir(mppy)_2_(2,9-diphen)]^+^ (mppy = 3-methyl-2-phenylpyridine, 2,9-diphen = 2,9-diphenyl-1,10-phenanthroline); Complex (**2**), [Ir(pbd)_2_(biq)]^+^ (pbd = 4-(pyridin-2-yl)benzaldehyde, biq = 2,2′-biquinoline); Complex (**3**), [Ir(ptpy)_2_(phen)]^+^ (ptpy = 2-(*p*-tolyl)pyridine, phen = 1,10-phenanthroline); Complex (**4**), [Ir(ppy)_2_(bpy)]^+^ (ppy = 2-phenylpyridine, bpy = 2,2′-bipyridine); Complex (**5**), [Ir(phq)_2_(dmobpy)]^+^ (phq = 2-phenylquinoline, dmobpy = 4,4′-dimethoxy-2,2′-bipyridine); Complex (**6**), [Ir(pbi)_2_(dmdpphen)]^+^ (pbi = 2-phenyl-1*H*-benzo[*d*]imidazole, dmdpphen = 2,9-dimethyl-4,7-diphenyl-1,10-phenanthroline).

**Figure 3:**
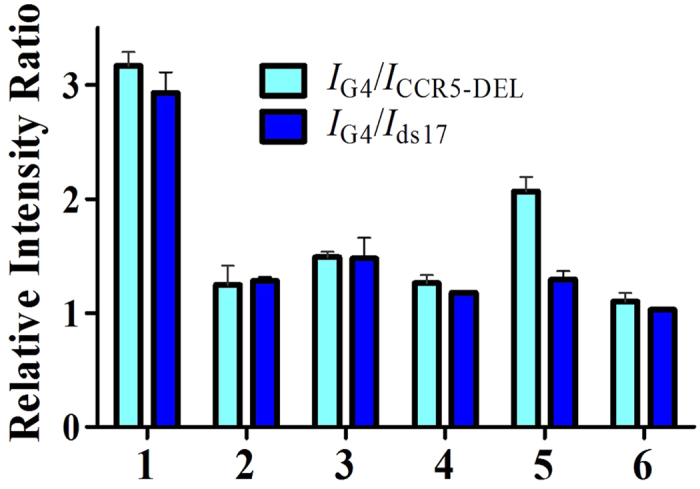
Diagrammatic bar array representation of the luminescence enhancement selectivity ratio of complexes (**1**–**6**) for G-quadruplex DNA (ON2) over dsDNA (ds17) and ssDNA (CCR5-DEL). Error bars represent the standard deviations of the results from three independent experiments.

In order to obtain a superior G-quadruplex-selective probe, six Ir(III) complexes **1**–**6** carrying different C^N and N^N ligands were synthesized in the present study (Fig. 2). Complexes **1**–**6** were initially tested for their ability to discriminate G-quadruplex (G4) from double-stranded DNA (dsDNA) and single-stranded DNA (ssDNA) (Table S1). Interestingly, the novel complex **1** [Ir(mppy)_2_(2,9-diphen)]^+^ (where mppy = 3-methyl-2-phenylpyridine, 2,9-diphen = 2,9-diphenyl-1,10-phenanthroline) showed an excellent discrimination for G-quadruplex DNA, with the highest *I*_G4_/*I*_dsDNA_ and *I*_G4_/*I*_ssDNA_ values among the six complexes tested (Fig. 3). Complex **1** also exhibited the strongest luminescence response to the ON2 G-quadruplex (*ca*. 3.6-fold), while only slight luminescence changes were observed for ssDNA (CCR5-DEL) and dsDNA (ds17) (Figure S1a and S1g). On the other hand, complexes **2**–**6** showed little or no selectivity towards G-quadruplex DNA (Figure S1b–S1f and S1g). Complex **1** possesses two phenyl groups at the 2 and 9 positions of its 1,10-phenanthroline N^N ligand, and one 3-methyl group on its 2-phenylpyridine C^N ligands. Complexes **2**, **4** and **6** showed the lowest selectivity to G-quadruplex DNA, suggesting that their N^N and C^N ligands were of an unsuitable structure to interact effectively with G-quadruplex DNA. These results suggest that the selectivity of the iridium(III) complexes for G-quadruplex DNA are dependent on their molecular structure, including substitution pattern and molecular size.

**Figure 4:**
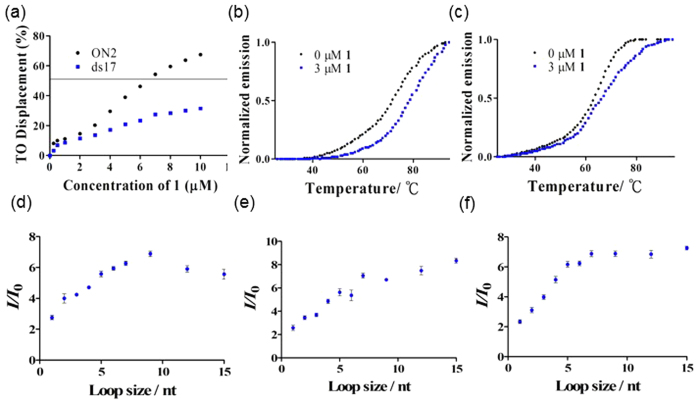
(**a**) G4-FID titration curves of DNA duplex ds17 (0.25 μM) and G-quadruplex ON2 (0.25 μM) in the presence of increasing concentration of complex **1** in Tris-HCl buffer. (^G4^DC_50_ value is determined by the half-maximal concentration of compound required to displace 50% TO from G-quadruplex DNA). (**b**) Melting profile of F21T G-quadruplex DNA (0.2 μM) in the absence and presence of **1** (3 μM). (**c**) Melting profile of F10T dsDNA (0.2 μM) in the absence and presence of **1** (3 μM). (**d**) Luminescence enhancement of complex **1** (1 μM) as a function of 5′-side loop, 5′-G_3_T_n_G_3_T_3_G_3_T_3_G_3_-3′. (**e**) Luminescence enhancement of complex **1** (1 μM) as a function of central loop, 5′-G_3_T_3_G_3_T_n_G_3_T_3_G_3_-3′ and (**f**) Luminescence enhancement of complex **1** (1 μM) as a function of 3′-side loop, 5′-G_3_T_3_G_3_T_3_G_3_T_n_G_3_-3′ (in nucleotides).

The characterization and photophysical properties of the complex **1** are presented in the ESI (Table S2 and Figure S2). The specific binding of complex **1** towards G-quadruplex DNA was further confirmed by G-quadruplex fluorescent intercalator displacement (G4-FID) experiments and fluorescence resonance energy transfer (FRET)-melting assays. The G4-FID assay showed that **1** was able to displace thiazole orange (TO) from the ON2 G-quadruplex with a ^G4^DC_50_ value (half-maximal concentration of compound required to displace 50% TO from DNA) of 7.0 μM (Fig. 4a). By contrast, less than 50% of TO was displaced from dsDNA even at the highest concentrations of **1** tested. The melting temperature (Δ*T*_m_) of the F21T G-quadruplex was increased by about 7.0 °C in the presence of **1** (Fig. 4b). On the other hand, **1** increased the melting temperature of F10T dsDNA by only 3.0 °C under the same conditions (Fig. 4c). Taken together, these results indicate that complex **1** selectively binds to G-quadruplex DNA over dsDNA or ssDNA. Since the signal transducer ON2 contains a long loop that is partially complementary with the adenosine aptamer ON1, the role of G-quadruplex loops in the binding interaction with complex **1** was investigated. The luminescence of **1** in the presence of different G-quadruplex DNA structures, including sequences containing a 5′-side loop, a central loop or a 3′-side loop, with loop sizes ranging from 1 to 15 nucleotides (nt) were tested (Fig. 4d–f). The results showed that the luminescence intensity of complex **1** generally increased with loop size regardless of the site of the loop. The luminescence enhancement of complex **1** increased from 2.7 to 6.9-fold for the 5′-side loop, from 2.4 to 8.3-fold for the central loop and from 2.3 to 7.2-fold for the 3′-side loop, as the loop size increased from 1 to 15 nt. This result shows that the G-quadruplex loop may play an important role in the interaction of G-quadruplex-complex **1**, and the loop size could affect the binding interaction between ligands and G-quadruplex DNA^52^. Based on these results and previously reported adenosine aptamer DNA sequences^11^,^37^, the 12 nt central loop was employed for the capture DNA ON2.

We first investigated the luminescence response of the system to adenosine (both Route A and B). Upon incubation with adenosine, the luminescence of **1** was significantly enhanced. We hypothesize that the luminescence enhancement of the system was due to the association of adenosine and aptamer ON1, which allows free ON2 to form a G-quadruplex motif that is recognized by **1**. To confirm that the observed luminescence enhancement arose from the formation of the ON2 G-quadruplex, a number of control experiments were carried out. No significant signal enhancement was observed when adenosine was added to **1** in the absence of ON1 and ON2, indicating that **1** did not interact with adenosine directly (Figure S3). We also designed a mutant DNA substrate ON2_m_ (5′-***A***_2_GT_3_***C***_2_GACTC_5_AG_2_TG***C***_2_T_3_G***A***_2_-3′, the bold italic bases are mutant bases) that is unable to form into a G-quadruplex structure due to the absence of key guanine residues in the two split G-quadruplex-forming sequences. As expected, for the mutant DNA substrate, only minimal enhancement in the luminescence of **1** was observed at 80 μM adenosine, indicating that the formation of the G-quadruplex motif is important for the mechanism of this detection platform (Figure S4). Circular dichroism (CD) spectroscopy was also employed to confirm the G-quadruplex conformational transition induced by adenosine (Figure S5). The CD spectrum of the ON1/ON2 reaction system in the absence of adenosine exhibits a positive peak at 260 nm and a negative peak at 235 nm, which are typical peaks for duplex DNA. Upon the addition of adenosine, the spectrum changes to reveal a positive band around 285 nm and a weak negative peak at 240 nm, consistent with the reported spectrum for an anti-parallel G-quadruplex^53^. Taken together, these results indicate that the luminescence enhancement of the system originated from the specific interaction between complex **1** with the ON2 G-quadruplex induced by the addition of adenosine.

**Figure 5:**
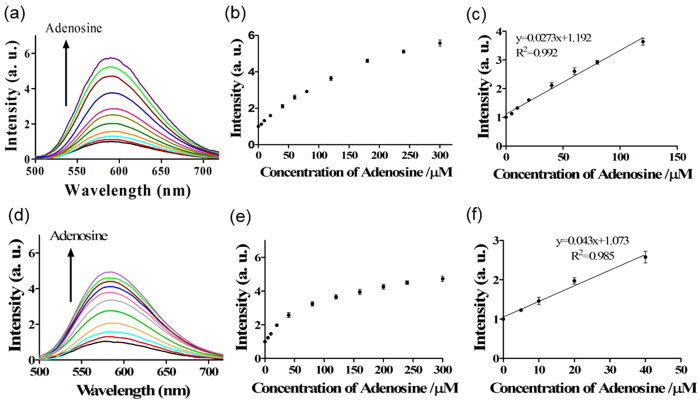
(**a**) Luminescence spectra of the system (Route A) in response to various concentrations of adenosine: 0, 5, 10, 20, 40, 60, 80, 120, 180, 240 and 300 μM. (**b**) The relationship between luminescence intensity at λ = 590 nm and adenosine concentration (Route A). (**c**) Linear plot of the change in luminescence intensity at λ = 590 nm *vs*. adenosine concentration (Route A). Error bars represent the standard deviations of the results from three independent experiments. (**d**) Luminescence spectra of the system (Route B) in response to various concentrations of adenosine: 0, 5, 10, 20, 40, 60, 80, 120, 180, 240 and 300 μM. (**e**) The relationship between luminescence intensity at λ = 590 nm and adenosine concentration for (Route B). (**f**) Linear plot of the change in luminescence intensity at λ = 590 nm *vs*. adenosine concentration (Route B). Error bars represent the standard deviations of the results from three independent experiments.

The mole ratio of ON1 and ON2 was fixed at 1:1 since ON2 was expected to hybridize to ON1 completely in the absence of adenosine. After optimization of the concentrations of ON1/ON2 (Figure S6) and complex **1** (Figure S7), the luminescence response of the system to different concentrations of adenosine (0 to 300 μM) was tested. For Route A, the system exhibited a *ca*. 5.7-fold enhancement in emission intensity at [adenosine] = 300 μM (Fig. 5a,b), with a linear range of detection for adenosine from 5 to 120 μM (y = 0.027x + 1.192, R^2^ = 0.992) (Fig. 5c). For Route B, the system showed a *ca*. 4.6-fold enhancement in emission intensity at [adenosine] = 300 μM (Fig. 5d,e), with a narrower linear range of 5 to 60 μM (y = 0.043x + 1.073, R^2^ = 0.985) (Fig. 5f) for Route B. These results suggest that Route A is preferable to Route B, which could be attributed to the fact that in Route A, the more stable adenosine-ON1 complex is formed first, which could limit the dissociation of this complex by ON2^37^. On the other hand, in Route B, the ON1-ON2 duplex is initially generated by a slow annealing process, and thus the desired dissociation of the duplex by adenosine may be incomplete. The detection limit of this assay for adenosine was estimated to be 5 μM at a signal-to-noise ratio (S/N) of 3 (Figure S8a and S8b) for both Route A and B. This detection limit is comparable to those of previously reported aptamer-based label-free methods^12^,^32^ (Table S3).

**Figure 6:**
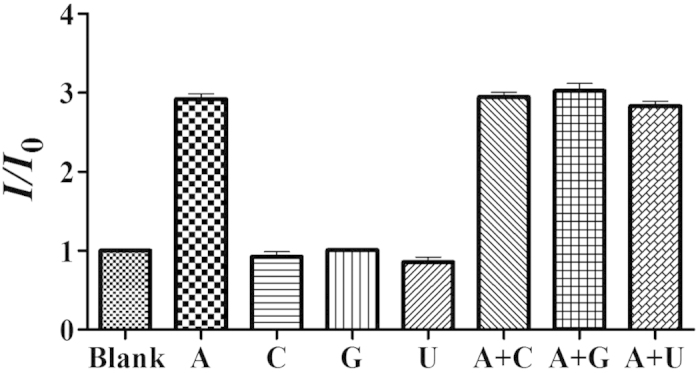
Selectivity of the G-quadruplex-based detection assay for adenosine over other nucleosides. The concentration of adenosine was 80 μM and the concentrations of the other nucleosides were 200 μM. Error bars represent the standard deviations of the results from three independent experiments.

The selectivity of this detection platform for adenosine was evaluated by investigating the luminescent response of the system to other nucleosides, such as cytidine (C), guanosine (G) and uridine (U). The results showed that only adenosine could significantly enhance the luminescence of the complex **1**/ON1/ON2 system (Fig. 6), while no signal enhancement with the other nucleosides was observed. These results indicate that the system displays significant selectivity for adenosine over other nucleosides, which is attributed to the specific interaction of adenosine towards the adenosine aptamer.

To evaluate the robustness of the detection system, we investigated the performance of the sensing platform for adenosine in the presence of cellular debris. In a reaction system containing 0.5% (*v*/*v*) cell extract, the complex **1**/ON1/ON2 DNA system (Route A) experienced a gradual increase in luminescence intensity as the concentration of adenosine was increased (Figure S9a–S9c). This result demonstrates that this assay could potentially be further developed as a sensitive adenosine detection method for biological sample analysis.

## Discussion

In summary, the novel luminescent Ir(III) complex **1** has been found to display excellent selectivity for G-quadruplex DNA over dsDNA and ssDNA, and was utilized to construct a label-free G-quadruplex-based detection platform for adenosine. In the absence of adenosine, the free aptamer ON1 is captured by the complementary capture DNA ON2, forming a duplex structure that interacts weakly with **1**. However, the luminescence of the system is significantly enhanced by the addition of adenosine due to the formation of adenosine-ON1 complexes, allowing free ON2 to fold into a G-quadruplex motif that is recognized by complex **1**. Furthermore, this assay was highly selective for adenosine over other nucleosides. Recently, we have reported a detection platform for adenosine-5′-triphosphate (ATP) utilizing the [Ir(ppy)_2_(biq)]PF_6_ (where ppy = 2-phenylpyridine, biq = 2,2′-biquinoline) as luminescent probe to monitor the G-quadruplex formation induced by ATP. The sensitivity of this detection platform (detection limit = 2.5 μM) is superior to that of the previously reported ATP detection method by using organic dye crystal violet (CV) (detection limit = 5.0 μM)^51^. More recently, an Ochratoxin A (OTA) detection method by using a similar iridium(III) complex [Ir(piq)_2_(tmphen)]^+^ (where piq = 1-phenylisoquinoline, tmphen = 3,4,7,8-tetramethyl-1,10-phenanthroline) was developed by our group, and this G-quadruplex-based luminescent switch-on assay was able to detect down to 5 nM of OTA^48^. In this study, complex **1** can monitor as low as 5 μM of adenosine, which is comparable to other reported label-free G-quadruplex-based assays for small molecules detection by using iridium(III) complexes. Overall, this label-free G-quadruplex-based luminescence switch-on platform is simple, rapid and cost-effective compared to conventional chromatographic or label-based assays, and can be readily adapted for the detection of other analytes by modification of the aptamer sequence. Thus, this simple technique can offer a new direction in the design of label-free G-quadruplex-based biosensors for the sensitive and selective detection of a wide spectrum of targets.

## Methods

### Chemicals and materials

Adenosine (A), cytidine (C), guanosine (G) and uridine (U) were purchased from Sigma Aldrich (St. Louis, MO) and were used as received. Other reagents, unless specified, were also purchased from Sigma Aldrich (St. Louis, MO). Iridium chloride hydrate (IrCl_3_·xH_2_O) was purchased from Precious Metals Online (Australia). All oligonucleotides were synthesized by Techdragon Inc. (Hong Kong, China).

### FRET melting assay

The ability of **1** to stabilize G-quadruplex DNA or dsDNA was investigated using a fluorescence resonance energy transfer (FRET) melting assay. The experimental procedure was similar to previously described^54^.

### G-quadruplex fluorescent intercalator displacement (G4-FID) assay

The G4-FID experiment was used to evaluate the binding affinity of **1** to G-quadruplex DNA or dsDNA. The experiment procedure was the same as previously reported^55^.

### Detection of adenosine in buffered solution

The adenosine aptamer ON1 (100 μM) and the capture DNA containing two split G-quadruplex-forming sequences ON2 (100 μM) were separately heated to 95 °C for 10 min, and then immediately immersed into 4 °C water to generate single-stranded oligonucleotides. The DNA was stored at –20 °C before use. As shown in Scheme 1, two different experimental routes for the assay were employed.

Route A: 0.5 μM of aptamer ON1 was incubated with various concentrations of adenosine in 500 μL Tris-HCl buffer (20 mM Tris-HCl, 100 mM NaCl, 10 mM MgCl_2_, pH 7.4) at 37 °C for 1 h, leading to the formation of an adenosine-aptamer complex. Then, the capture DNA probe ON2 (0.5 μM) was added to the reaction mixture to hybridize with the non-reacted aptamer ON1. After 1 h’s incubation at 37 °C, 75 mM of potassium ions were added to induce the formation of unhybridized ON2 into a G-quadruplex structure.

Route B: 0.5 μM of ON1 was hybridized with 0.5 μM of the capture DNA ON2 by annealing at 95 °C for 10 min, cooling to room temperature at 0.1 °C/s, and further incubation at room temperature for 1 h to ensure formation of the duplex substrate. The mixture was then reacted with various concentrations of adenosine in 500 μL Tris-HCl buffer (20 mM Tris-HCl, 100 mM NaCl, 10 mM MgCl_2_, 75 mM KCl, pH 7.4) at 37 °C for 1 h.

For both routes, 1 μM of complex **1** was added to the mixture after the final step. Emission spectra were recorded in the 500–750 nm range using an excitation wavelength of 310 nm. For the detection of adenosine in cell extract, 0.5 μM of aptamer ON1 was first incubated with different concentrations of adenosine in 500 μL Tris-HCl buffer (20 mM Tris-HCl, 100 mM NaCl, 10 mM MgCl_2_, pH 7.4) containing 0.5% (v/v) cell extract at 37 °C for 1 h, leading to the formation of an adenosine-aptamer complex. The following steps are the same as described for adenosine detection in buffered solution, Route A.

### Synthesis

Complex **1** was prepared according to (modified) literature methods. The precursor iridium(III) complex dimer [Ir_2_(mppy)_4_Cl_2_] was prepared as reported^56^. Then, a suspension of [Ir_2_(mppy)_4_Cl_2_] (0.1 mmol) and the corresponding N^N ligand (2,9-diphen) (0.22 mmol) in a mixture of DCM:methanol (1:1, 20 mL) was refluxed overnight under a nitrogen atmosphere. The work-up procedure was the same as previously reported^57^. Complex **1** was characterized by ^1^H-NMR, ^13^C-NMR, high resolution mass spectrometry (HRMS) and elemental analysis.

Complex **1**. 72%. ^1^H NMR (400 MHz, acetonitrile-*d*_3_) δ 8.70 (d, *J* = 8.0 Hz, 2 H), 8.27 (s, 2 H), 7.88 (dd, *J* = 5.6, 1.2 Hz, 2 H), 7.65 (d, *J* = 8.0 Hz, 2 H), 7.61 (dd, *J* = 7.6, 0.8 Hz, 2 H), 7.42 (d, *J* = 8.0 Hz, 2 H), 7.09–7.04 (m, 2 H), 6.85 (dd, *J* = 7.6 , 5.6 Hz, 2 H), 6.75 (t, *J* = 7.6 Hz, 4 H), 6.63 (d, *J* = 7.2 Hz, 4 H), 6.54–6,50 (m, 2 H), 6.17–6.13 (m, 2 H), 5.40 (dd, *J* = 8.0, 1.2 Hz, 2 H), 2.7 (s, 6 H); ^13^C NMR (100 MHz, acetonitrile-*d*_3_) δ 165.6, 165.4, 148.9, 148.3, 148.0, 143.6, 141.4, 138.6, 138.0, 131.9, 130.5, 130.1, 128.6, 127.7, 127.6, 127.6, 127.4, 126.9, 126.8, 120.6, 120.0, 22.1; MALDI-TOF-HRMS: Calcd. for C_48_H_36_IrN_4_ [M–PF_6_]^+^ : 861.2569; Found: 861.2521. Anal.: (C_48_H_36_IrN_4_PF_6_ + H_2_O) C, H, N: Calcd.: 56.30, 3.74, 5.47; Found: 56.67, 3.71, 5.59.

Complex **2**. Reported^58^.

Complex **3**. Reported^59^.

Complex **4**. Reported^60^.

Complexes **5** and **6**. Reported^61^.

## Additional Information

**How to cite this article**: Lu, L. *et al*. Development of a luminescent G-quadruplex-selective iridium(III) complex for the label-free detection of adenosine. *Sci. Rep*. **6**, 19368; doi: 10.1038/srep19368 (2016).

## Supplementary Material

Supplementary Information
