# Supplementary material for: Development of a luminescent G-quadruplex-selective iridium(III) complex for the label-free detection of adenosine
Source: Sci Rep. 2016 Jan 18;6:19368. doi: 10.1038/srep19368 (PMC4726048; doi:10.1038/srep19368)
Supplement: Supplementary Information [file srep19368-s1.doc]

**Supporting Information**

**Development of a luminescent G-quadruplex-selective iridium(III) complex for the label-free detection of adenosine**

Lihua Lu^1,§^, Hai-Jing Zhong^2,§^, Bingyong He^1^, Chung-Hang Leung*^,2^ and Dik-Lung Ma*^,1,3^

^1^Department of Chemistry, Hong Kong Baptist University, Kowloon Tong, Hong Kong, China. E-mail: [edmondma@hkbu.edu.hk](mailto:edmondma@hkbu.edu.hk). ^2^State Key Laboratory of Quality Research in Chinese Medicine, Institute of Chinese Medical Sciences, University of Macau, Macao, China. E-mail: [duncanleung@umac.mo](mailto:duncanleung@umac.mo). ^3^Partner State Key Laboratory of Environmental and Biological Analysis, Hong Kong Baptist University, Hong Kong, China.

§ These authors contribute equally to this work.

**Experimental methods**

**General experimental.** Mass spectrometry was performed at the Mass Spectroscopy Unit at the Department of Chemistry, Hong Kong Baptist University, Hong Kong (China). Deuterated solvents for NMR purposes were obtained from Armar and used as received. Circular dichroism (CD) spectra were collected on a JASCO-815 spectrometer.

^1^H and ^13^C NMR were recorded on a Bruker Avance 400 spectrometer operating at 400 MHz (^1^H) and 100 MHz (^13^C). ^1^H and ^13^C chemical shifts were referenced internally to solvent shift (acetonitrile-*d*_3_: ^1^H, δ1.94, ^13^C, δ118.7). Chemical shifts (δ) are quoted in ppm, the downfield direction being defined as positive. Uncertainties in chemical shifts are typically ± 0.01 ppm for ^1^H and ± 0.05 for ^13^C. Coupling constants are typically ± 0.1 Hz for ^1^H-^1^H and ± 0.5 Hz for ^1^H-^13^C couplings. The following abbreviations are used for convenience in reporting the multiplicity of NMR resonances: s, singlet; d, doublet; t, triplet; q, quartet; m, multiplet; br, broad. All NMR data was acquired and processed using standard Bruker software (Topspin).

**Stock solution preparation.** The stock solutions of Ir(III) complexes were prepared in acetonitrile with a concentration of 1 mM. Adenosine, cytidine, guanosine and uridine were dissolved in Milli Q water at an initial concentration of 10 mM.

**Photophysical measurement.** Emission spectra, absorbance, lifetime measurements and Luminescence quantum yields were determined according to a reference.^1^

**Luminescence response of iridium(III) complexes towards different forms of DNA.** The G-quadruplex DNA-forming sequences ON2 was annealed in Tris-HCl buffer (20 mM Tris, 100 mM KCl, pH 7.4) and were stored at –20 °C before use. Complexes **1**–**6** (1 µM) was added to 5 µM of ssDNA, dsDNA or ON2 G-quadruplex DNA in Tris-HCl buffer (20 mM Tris-HCl, pH 7.4), then their emission intensity were tested.

**Total cell extract preparation.** The TRAMPC1 (ATCC® CRL2730™) cell line was purchased from American Type Culture Collection (Manassas, VA 20108 USA). Prostate cancer cells were trypsinized and resuspended in TE buffer (10 mM Tris–HCl 7.4, 1 mM EDTA). After incubation on ice for 10 min, the lysate was centrifuged and the supernatant was collected.

**Table S1.** DNA sequences used in this project:

| DNA | Sequence |
| --- | --- |
| ON1 | 5′-AC_2_TG_5_AGTAT_2_GCG_2_AG_2_A_2_G_2_T-3′ |
| ON2 | 5′- G_3_T_3_G_3_ACTC_5_AG_2_TG_3_T_3_G_3_-3′ |
| CCR5-DEL | 5′-CTCAT_4_C_2_ATACAT_2_A_3_GATAGTCAT-3′ |
| ds17 | 5′-C_2_AGT_2_CGTAGTA_2_C_3_-3′  5′-G_3_T_2_ACTACGA_2_CTG_2_-3′ |
| F21T | 5′-FAM-(G_3_[T_2_AG_3_]_3_)-TAMRA-3′ |
| F10T | 5′-FAM-TATAGCTA-HEG-TATAGCTATAT-TAMRA-3′ |
| ON2_m_ | 5′- ***A***_2_GT_3_***C***_2_G ACTC_5_AG_2_TG***C***_2_T_3_G***A***_2_-3′ |

1. The bold italic bases are mutant bases.

**Table S2** Photophysical properties of iridium(III) complex **1**.

| Complex | Quantum yield | λ_em_ / nm | Life time / µs | UV/vis absorption  λ_abs_ / nm (ε/ dm^3^mol^–1^cm^–1^) |
| --- | --- | --- | --- | --- |
| **1** | 0.158 | 590 | 4.39 | 218 (1.92 × 10^5^), 257 (1.15 × 10^5^), 338 (1.43 × 10^4^) |

**Table S3** Comparison of aptamer-based adenosine detection assays reported in recent years.

| Method | Selectivity | Detection limit | Reference | Labeled DNA? |
| --- | --- | --- | --- | --- |
| A luminescent G-quadruplex-selective iridium(III) complex for the label-free detection of adenosine | Discriminate adenosine from its analogues | 5 µM | Our work | No |
| A turn-on fluorescent aptasensor for adenosine detection based on split aptamers and graphene oxide | Discriminate adenosine from its analogues | 6 µM | 2 | Yes |
| Detection of adenosine using surface-enhanced raman scattering based on structure-switching signaling aptamer | Discriminate adenosine from its analogues | 0.01 µM | 3 | Yes |
| A sensitive aptasensor for adenosine based on the quenching of Ru (bpy)_3_^2+^-doped silica nanoparticle ECL by ferrocene | Discriminate adenosine from its analogues | 0.031 nM | 4 | Yes |
| Hairpin assembly circuit-based fluorescence cooperative amplification strategy for enzyme-free and label-free detection of adenosine | Discriminate adenosine from its analogues | 0.97 µM | 5 | No |
| Label-free electrochemical detection of nanomolar adenosine based on target-induced aptamer displacement | Discriminate adenosine from its analogues | 1 nM | 6 | No |
| A novel enzyme-free and label-free fluorescence aptasensor for amplified detection of adenosine | Discriminate adenosine from its analogues | 6 µM | 7 | No |
| Aptamer-functionalized hydrogel microparticles for fast visual detection of adenosine | --------- | 50 µM | 8 | No |
| An ultrasensitive fluorescent aptasensor for adenosine detection based on exonuclease III assisted signal amplification | Discriminate adenosine from its analogues | 1 nM | 9 | Yes |
| Time-resolved fluorescence biosensor for adenosine detection based on home-made europium complexes | Discriminate adenosine from its analogues | 5.61 nM | 10 | Yes |
| Flow cytometry-assisted detection of adenosine in serum with an immobilized aptamer sensor | Discriminate adenosine from its analogues | 178 µM | 11 | Yes |
| Au–Ag core–shell nanoparticles with controllable shell thicknesses for the detection of adenosine by surface enhanced Raman scattering | --------- | 1 nM | 12 | Yes |
| Adenosine detection by using gold nanoparticles and designed aptamer sequences | Discriminate adenosine from its analogues | 250 µM | 13 | No |
| Direct detection of adenosine in undiluted serum using a luminescent aptamer sensor attached to a terbium complex | Discriminate adenosine from its analogues | 60 µM | 14 | Yes |
| KF polymerase-based fluorescence aptasensor for the label-free adenosine detection | Discriminate adenosine from its analogues | 12 µM | 15 | No |
| Aptamer‐based origami paper analytical device for electrochemical detection of adenosine | --------- | 11.8 µM | 16 | Yes |
| Fast colorimetric sensing of adenosine based on a general sensor design involving aptamers and nanoparticles | --------- | 300 µM^a^ | 17 | Yes |
| Highly sensitive, reusable electrochemical aptasensor for adenosine | Discriminate adenosine from its analogues | 16.5 pM | 18 | Yes |
| Rational design of an optical adenosine sensor by conjugating a DNA aptamer with split DNAzyme halves | Discriminate adenosine from its analogues | 6 µM | 19 | No |
| An aptazyme-based electrochemical biosensor for the detection of adenosine | Discriminate adenosine from its analogues | 5 nM | 20 | No |
| A novel aptasensor for the detection of adenosine in cancer cells by electrochemiluminescence of nitrogen doped TiO2 nanotubes | Discriminate adenosine from its analogues | 10 nM | 21 | No |
| Methylene blue as an indicator for sensitive electrochemical detection of adenosine based on aptamer switch | Discriminate adenosine from its analogues | 0.01 µM | 22 | No |
| Adenosine–aptamer recognition-induced assembly of gold nanorods and a highly sensitive plasmon resonance coupling assay of adenosine in the brain of model SD rat | Discriminate adenosine from its analogues | 2.0 nM | 23 | No |
| A solid-state electrochemiluminescence sensing platform for detection of adenosine based on ferrocene-labeled structure-switching signaling aptamer | Discriminate adenosine from its analogues | 5 nM | 24 | Yes |
| Reusable electrochemical sensing platform for highly sensitive detection of adenosine based on structure-switching signaling aptamers | Discriminate adenosine from its analogues | 20 nM | 25 | Yes |
| Abasic site-containing DNAzyme and aptamer for label-free fluorescent detection of adenosine with high sensitivity, selectivity, and tunable dynamic range | --------- | 3.4 µM | 26 | No |
| Aptamer-based electrochemical biosensor for label-free voltammetric detection of adenosine | Discriminate adenosine from its analogues | 10 nM | 27 | No |
| Label-free aptamer-based chemiluminescence detection of adenosine | Discriminate adenosine from its analogues | 0.08 µM | 28 | No |
| DNA aptamer folding on magnetic beads for sequential detection of adenosine by substrate-resolved chemiluminescence technology | --------- | 5.2 nM | 29 | Yes |
| A one-step sensitive dynamic light scattering method for adenosine detection using split aptamer fragments | Discriminate adenosine from its analogues | 7 nM | 30 | No |
| Electrogenerated chemiluminescence detection of adenosine based on triplex DNA biosensor | Discriminate adenosine from its analogues | 0.27 nM | 31 | Yes |
| A gold nanoparticles-modified aptamer beacon for urinary adenosine detection based on structure-switching/ fluorescence-“turning on” mechanism | Discriminate adenosine from its analogues | 6 nM | 32 | Yes |
| Electrochemical biosensor for detection of adenosine based on structure-switching aptamer and amplification with reporter probe DNA modified Au nanoparticles | Discriminate adenosine from its analogues (Apart from Guanosine) | 0.18 nM | 33 | No |
| A multimode responsive aptasensor for adenosine detection | --------- | 10 µM^a^ | 34 | No |
| Simple and rapid colorimetric adenosine biosensors based on DNA aptamer and noncrosslinking gold nanoparticle aggregation | --------- | 10 µM | 35 | No |

1. The lowest detectable concentration.

**Figure S1.** (a–f) Luminescence response of complexes **1**–**6** (1 μM) in 20 mM Tris buffer (pH 7.4) in the presence of 5 µM ssDNA (CCR5-DEL), 5 µM dsDNA (ds17) and 5 µM G-quadruplex DNA (ON2), respectively. ON2 G-quadruplex DNA was pre-annealed in Tris buffer (20 mM, 100 mM KCl, pH 7.4). (g) Diagrammatic bar array representation of the luminescence enhancement selectivity of complexes **1**–**6** (1 μM) in 20 mM Tris buffer (pH 7.4) in the presence of 5 µM ssDNA (CCR5-DEL), 5 µM dsDNA (ds17) and 5 µM G-quadruplex (ON2), respectively. Error bars represent the standard deviations of the results from three independent experiments.


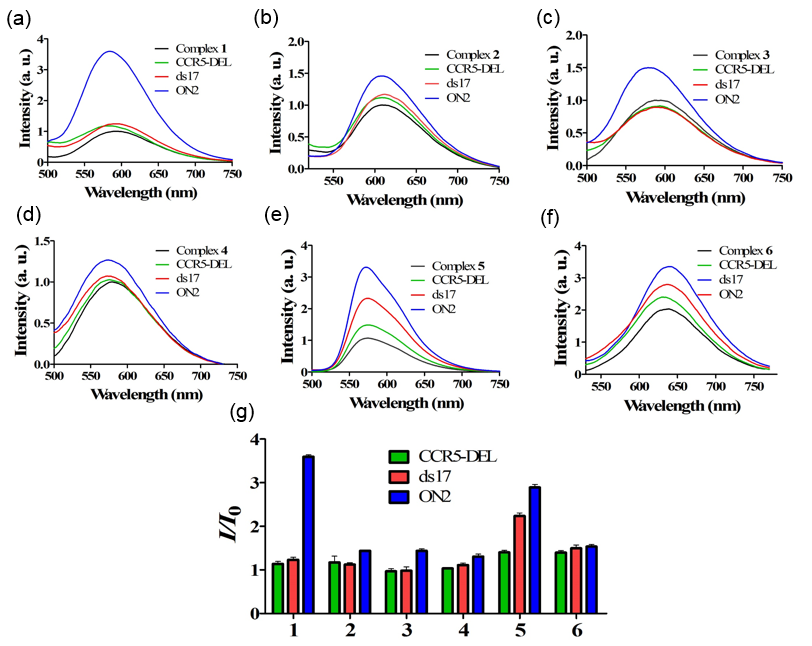


**Figure S2.** UV/vis absorption and normalized emission spectra of complex **1** (2.5 µM) in acetonitrile solution at 298 K.

**
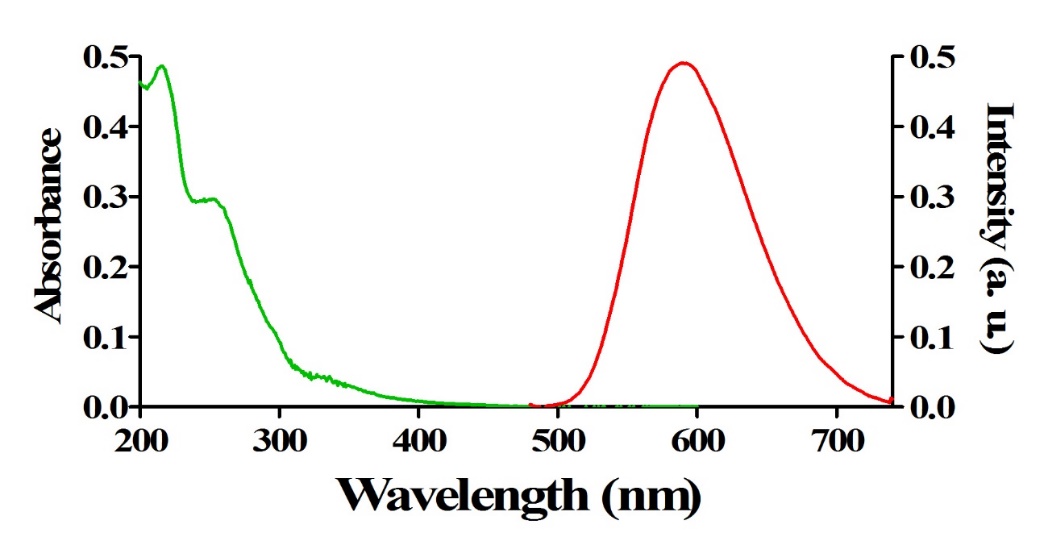
**

**Figure S3.** Luminescence response of the system with the complex alone ([complex **1**] = 1 µM) in the absence and presence of adenosine (500 µM).


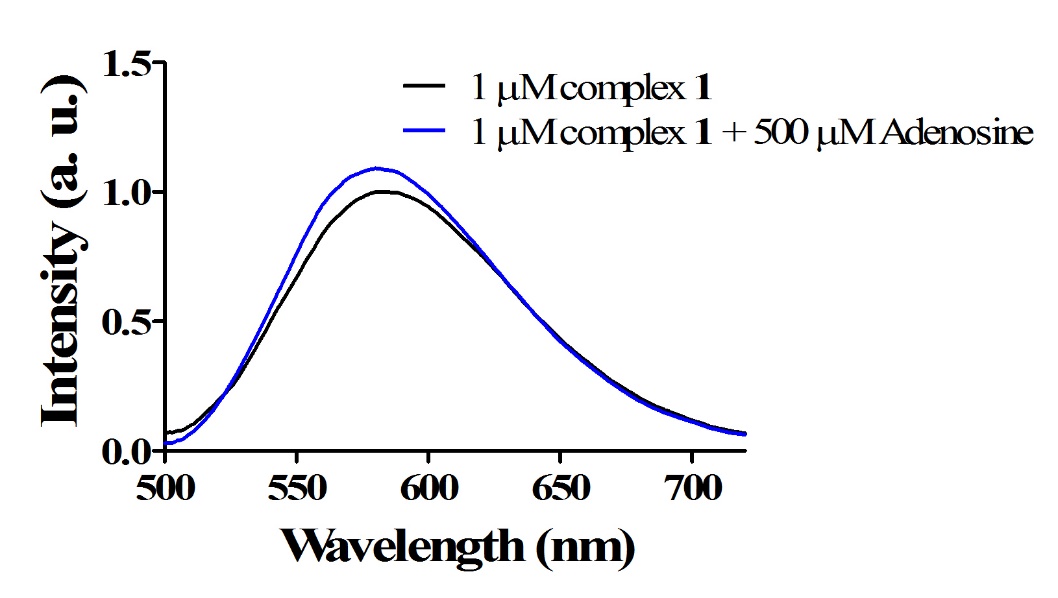


**Figure S4.** Relative luminescence response of complex **1** (1 μM) in the presence of wild- typed or mutant DNA. Experimental conditions: 0.5 μΜ of ON1 and 80 μM of adenosine were firstly incubated in Tris-HCl buffer (20 mM Tris, 100 mM NaCl, 10 mM MgCl_2_, pH 7.4) at 37 °C for 1 h, then 0.5 μM of ON2 or 0.5 μM of ON2_m_ and 75 mM KCl were added. Error bars represent the standard deviations of the results from three independent experiments.

**
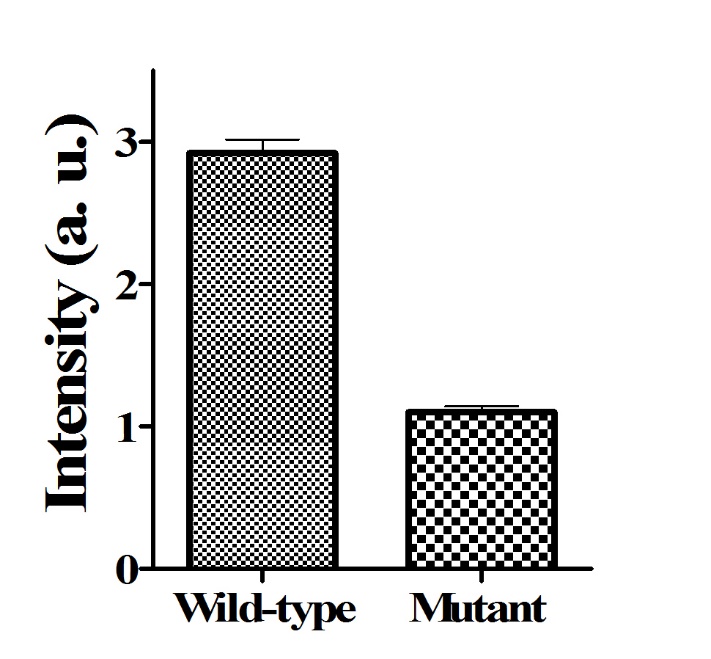
**

**Figure S5.** Circular dichroism (CD) spectrum of ON1 (2 μM) and ON2 (2 μM) in the absence (blank) or presence (blue) of 200 µM of adenosine recorded in Tris-HCl buffer (20 mM Tris-HCl, 100 mM NaCl, 10 mM MgCl_2_, pH 7.4). The processing procedure is the same as the Route A.

**
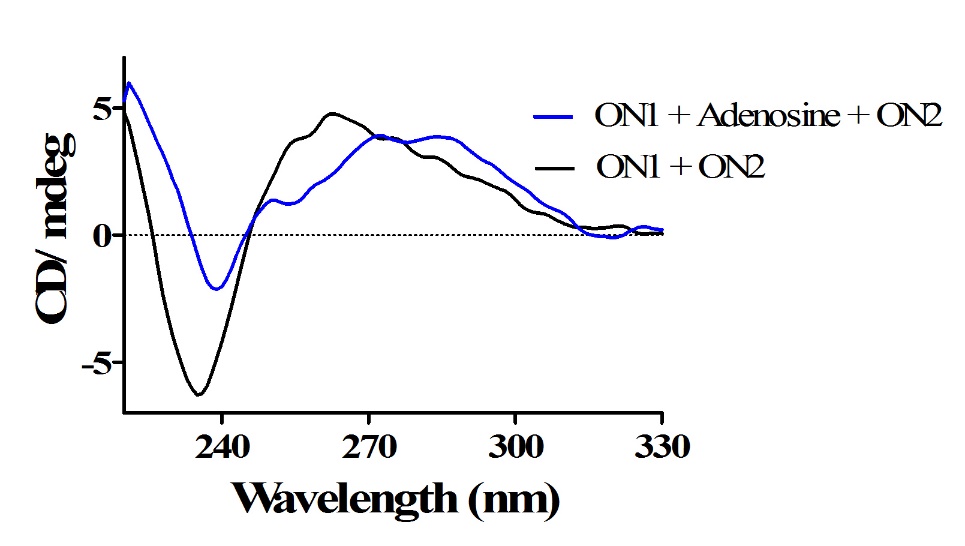
**

**Figure S6.** Relative luminescence response of the system in the absence or presence of adenosine (80 μM) at various concentrations of ON1 and ON2 (0.1, 0.25, 0.5, and 1 μM). Experimental conditions: certain amount of ON1 was firstly incubated with 80 μM of adenosine in Tris-HCl buffer (20 mM Tris-HCl, 100 mM NaCl, 10 mM MgCl_2_, pH 7.4) at 37 °C for 1 h, then the same amount of ON2 were added. Finally, 75 mM of K^+^ and 1 μM of complex **1** were added for emission testing. Error bars represent the standard deviations of the results from three independent experiments.


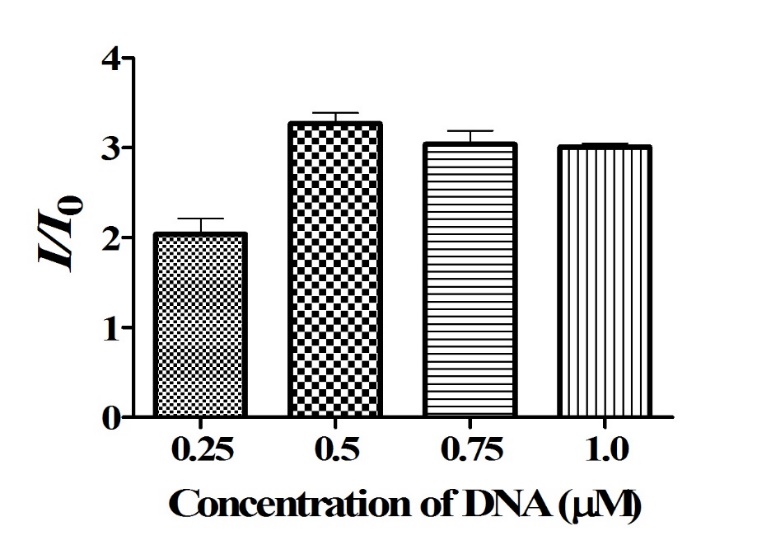


**Figure S7.** Relative luminescence response of the system in the absence or presence of adenosine (80 μM) at various concentrations of complex **1** (0.5, 0.75, 1.0, and 1.25 μM). Experimental conditions: 0.5 μM of ON1 was firstly incubated with adenosine/without adenosine in Tris-HCl buffer (20 mM Tris-HCl, 100 mM NaCl, 10 mM MgCl_2_, pH 7.4) at 37 °C for 1 h, then 0.5 μM of ON2 were added. Finally, 75 mM of K^+^ and certain amount of complex **1** were added for emission testing. Error bars represent the standard deviations of the results from three independent experiments.


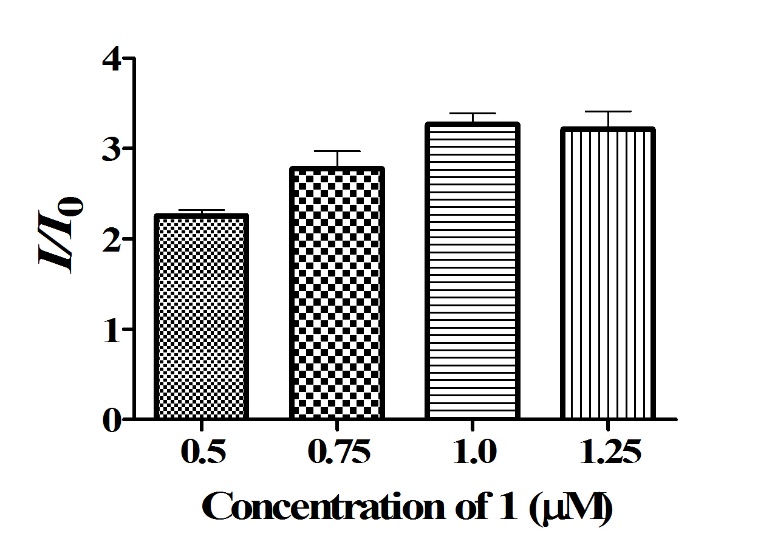


**Figure S8.** Emission spectral traces of complex **1** (1 μM), ON1 (0.5 μM) and ON2 (0.5 μM) in the presence/absence of adenosine (5 μM) in Tris-HCl buffer (20 mM Tris-HCl, 100 mM NaCl, 10 mM MgCl_2_, pH 7.4) for (a) Route A and (b) Route B.


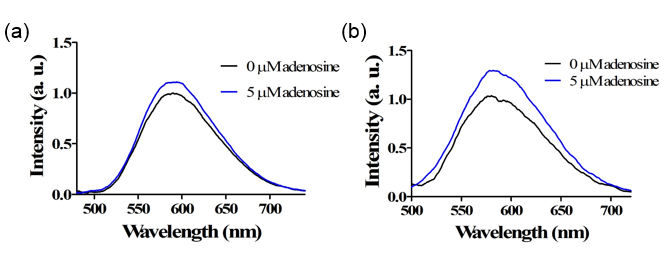


**Figure S9.** (a) Luminescence spectra of the complex **1**/ON1/ON2 system in a reaction system containing 0.5% (v/v) cell extract in response to various concentrations of adenosine: 0, 10, 20, 40, 60, 80, 120, 160, 200, 240, and 300 μM. (b) The relationship between luminescence intensity at λ = 590 nm and adenosine concentration. (c) Linear plot of the change in luminescence intensity at λ = 590 nm vs. adenosine concentration. Error bars represent the standard deviations of the results from three independent experiments.


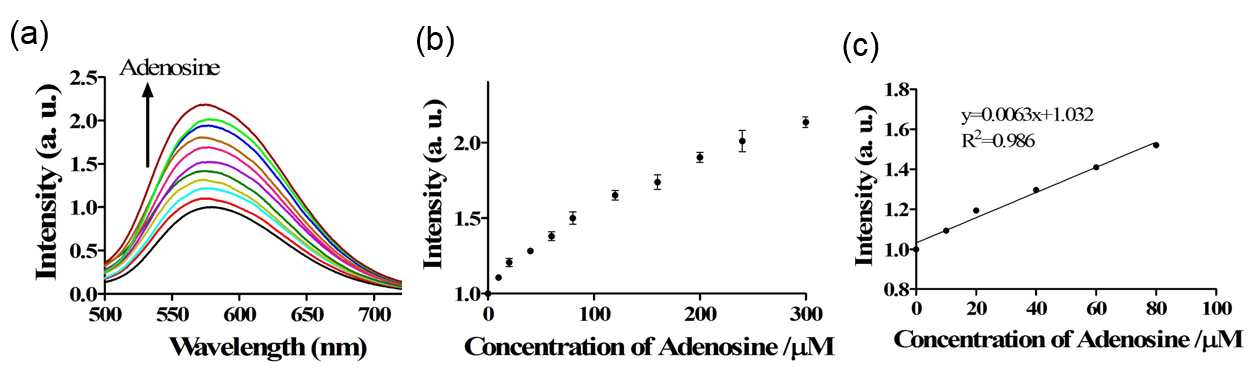


**Reference**

1 Lu, L. *et al.* Detection of nicking endonuclease activity using a G-quadruplex-selective luminescent switch-on probe. *Chem. Sci.* **5**, 4561-4568 (2014).

2 Bai, Y. *et al.* A turn-on fluorescent aptasensor for adenosine detection based on split aptamers and graphene oxide. *Analyst* **139**, 1843-1846 (2014).

3 Chen, J.-W. *et al.* Detection of adenosine using surface-enhanced Raman scattering based on structure-switching signaling aptamer. *Biosens. Bioelectron.* **24**, 66-71 (2008).

4 Chen, L. *et al.* A sensitive aptasensor for adenosine based on the quenching of Ru (bpy) 32+-doped silica nanoparticle ECL by ferrocene. *Chem. Commun.* **46**, 7751-7753 (2010).

5 Feng, C., Zhu, J., Sun, J., Jiang, W. & Wang, L. Hairpin assembly circuit-based fluorescence cooperative amplification strategy for enzyme-free and label-free detection of small molecule. *Talanta* (2015).

6 Feng, K. *et al.* Label-free electrochemical detection of nanomolar adenosine based on target-induced aptamer displacement. *Electrochem. Commun.* **10**, 531-535 (2008).

7 Fu, B., Cao, J., Jiang, W. & Wang, L. A novel enzyme-free and label-free fluorescence aptasensor for amplified detection of adenosine. *Biosens. Bioelectron.* **44**, 52-56 (2013).

8 Helwa, Y., Dave, N., Froidevaux, R., Samadi, A. & Liu, J. Aptamer-functionalized hydrogel microparticles for fast visual detection of mercury (II) and adenosine. *ACS Appl. Mater. Interfaces* **4**, 2228-2233 (2012).

9 Hu, P., Zhu, C., Jin, L. & Dong, S. An ultrasensitive fluorescent aptasensor for adenosine detection based on exonuclease III assisted signal amplification. *Biosens. Bioelectron.* **34**, 83-87 (2012).

10 Huang, D.-W., Niu, C.-G., Zeng, G.-M. & Ruan, M. Time-resolved fluorescence biosensor for adenosine detection based on home-made europium complexes. *Biosens. Bioelectron.* **29**, 178-183 (2011).

11 Huang, P.-J. J. & Liu, J. Flow cytometry-assisted detection of adenosine in serum with an immobilized aptamer sensor. *Anal. Chem.* **82**, 4020-4026 (2010).

12 Ko, F.-H., Tai, M. R., Liu, F.-K. & Chang, Y.-C. Au–Ag core–shell nanoparticles with controllable shell thicknesses for the detection of adenosine by surface enhanced Raman scattering. *Sens. Actuators B Chem.* **211**, 283-289 (2015).

13 Li, F. *et al.* Adenosine detection by using gold nanoparticles and designed aptamer sequences. *Analyst* **134**, 1355-1360 (2009).

14 Li, L.-L., Ge, P., Selvin, P. R. & Lu, Y. Direct detection of adenosine in undiluted serum using a luminescent aptamer sensor attached to a terbium complex. *Anal. Chem.* **84**, 7852-7856 (2012).

15 Liao, D., Jiao, H., Wang, B., Lin, Q. & Yu, C. KF polymerase-based fluorescence aptasensor for the label-free adenosine detection. *Analyst* **137**, 978-982 (2012).

16 Liu, H., Xiang, Y., Lu, Y. & Crooks, R. M. Aptamer‐based origami paper analytical device for electrochemical detection of adenosine. *Angew. Chem. Int. Edit* **124**, 7031-7034 (2012).

17 Liu, J. & Lu, Y. Fast colorimetric sensing of adenosine and cocaine based on a general sensor design involving aptamers and nanoparticles. *Angew. Chem. Int. Edit* **118**, 96-100 (2006).

18 Liu, Z. *et al.* Highly sensitive, reusable electrochemical aptasensor for adenosine. *Electrochim. Acta* **54**, 6207-6211 (2009).

19 Lu, N., Shao, C. & Deng, Z. Rational design of an optical adenosine sensor by conjugating a DNA aptamer with split DNAzyme halves. *Chem. Commun.*, 6161-6163 (2008).

20 Sun, C. *et al.* An aptazyme-based electrochemical biosensor for the detection of adenosine. *Anal. Chim. Acta* **669**, 87-93 (2010).

21 Tian, C.-Y., Xu, J.-J. & Chen, H.-Y. A novel aptasensor for the detection of adenosine in cancer cells by electrochemiluminescence of nitrogen doped TiO2 nanotubes. *Chem. Commun.* **48**, 8234-8236 (2012).

22 Wang, J., Wang, F. & Dong, S. Methylene blue as an indicator for sensitive electrochemical detection of adenosine based on aptamer switch. *J. Electroanal. Chem.* **626**, 1-5 (2009).

23 Wang, J. *et al.* Adenosine–aptamer recognition-induced assembly of gold nanorods and a highly sensitive plasmon resonance coupling assay of adenosine in the brain of model SD rat. *Analyst* **135**, 2826-2831 (2010).

24 Wang, X., Dong, P., He, P. & Fang, Y. A solid-state electrochemiluminescence sensing platform for detection of adenosine based on ferrocene-labeled structure-switching signaling aptamer. *Anal. Chim. Acta* **658**, 128-132 (2010).

25 Wu, Z.-S. *et al.* Reusable electrochemical sensing platform for highly sensitive detection of small molecules based on structure-switching signaling aptamers. *Anal. Chem.* **79**, 2933-2939 (2007).

26 Xiang, Y., Tong, A. & Lu, Y. Abasic site-containing DNAzyme and aptamer for label-free fluorescent detection of Pb2+ and adenosine with high sensitivity, selectivity, and tunable dynamic range. *J. Am. Chem. Soc.* **131**, 15352-15357 (2009).

27 Yan, F., Wang, F. & Chen, Z. Aptamer-based electrochemical biosensor for label-free voltammetric detection of thrombin and adenosine. *Sens. Actuators B Chem.* **160**, 1380-1385 (2011).

28 Yan, X., Cao, Z., Kai, M. & Lu, J. Label-free aptamer-based chemiluminescence detection of adenosine. *Talanta* **79**, 383-387 (2009).

29 Yan, X., Cao, Z., Lau, C. & Lu, J. DNA aptamer folding on magnetic beads for sequential detection of adenosine and cocaine by substrate-resolved chemiluminescence technology. *Analyst* **135**, 2400-2407 (2010).

30 Yang, X. *et al.* A one-step sensitive dynamic light scattering method for adenosine detection using split aptamer fragments. *Anal. Methods* **3**, 59-61 (2011).

31 Ye, S., Li, H. & Cao, W. Electrogenerated chemiluminescence detection of adenosine based on triplex DNA biosensor. *Biosens. Bioelectron.* **26**, 2215-2220 (2011).

32 Zhang, J.-Q. *et al.* A gold nanoparticles-modified aptamer beacon for urinary adenosine detection based on structure-switching/fluorescence-“turning on” mechanism. *J. Pharm. Biomed. Anal.* **70**, 362-368 (2012).

33 Zhang, S., Xia, J. & Li, X. Electrochemical biosensor for detection of adenosine based on structure-switching aptamer and amplification with reporter probe DNA modified Au nanoparticles. *Anal. Chem.* **80**, 8382-8388 (2008).

34 Zhao, N. *et al.* A Multimode Responsive Aptasensor for Adenosine Detection. *J. Nanomater.* **2014**, 1-7 (2014).

35 Zhao, W., Chiuman, W., Brook, M. A. & Li, Y. Simple and rapid colorimetric biosensors based on DNA aptamer and noncrosslinking gold nanoparticle aggregation. *ChemBioChem* **8**, 727-731 (2007).
